# Pulmonary sequestration presenting with left upper abdominal bloating and marked elevation of serum carbohydrate antigen 19-9: A case report

**DOI:** 10.3892/ol.2014.1960

**Published:** 2014-03-11

**Authors:** PING HAN, YI LUO, DEAN TIAN, WEI YAN, JINGMEI LIU, YING CHANG, HUAPING XIE, WANG WEI, HUANJUN HUANG

**Affiliations:** 1 Department of Gastroenterology, Tongji Hospital, Tongji Medical College, Huazhong University of Science and Technology, Wuhan, Hubei 430030, P.R. China; 2 Tuberculosis Prevention Institution of Wuhan, Wuhan, Hubei 430030, P.R. China

**Keywords:** carbohydrate antigen 19-9, pulmonary sequestration, abdominal bloating, immunohistochemistry

## Abstract

Carbohydrate antigen 19-9 (CA19-9) is widely accepted as a tumor marker for cancers of the biliary, pancreatic and gastrointestinal tracts. Occasionally, CA19-9 is markedly elevated in the serum of patients with benign diseases. Pulmonary sequestration is a rare malformation that is characterized by the presence of lung tissue with abnormal or absent communication with the bronchi, to which blood is supplied by the systemic arteries. The current study presents a 48-year-old male who presented with upper left abdominal bloating and marked elevation of serum CA19-9 levels. The patient was referred to the Tongji Hospital (Wuhan, China) with suspected hepato-biliary-pancreatic disease and, following surgery, was diagnosed with intralobar pulmonary sequestration. Immunohistochemistry showed marked positive staining for CA19-9 in the sequestrated lung tissue. The patient’s symptoms improved and the CA19-9 levels returned to the normal range following surgery. Therefore, the symptoms of upper left abdominal bloating and marked elevation of serum CA19-9 levels, in this case, may have resulted from the intralobar pulmonary sequestration.

## Introduction

Carbohydrate antigen 19-9 (CA19-9) is a sialyl Lewis A antigen and is widely used as a tumor marker for cancers of the biliary, pancreatic and gastrointestinal tracts (1–3). However, in benign diseases, such as pancreatitis, liver cirrhosis, biliary diseases and diabetes mellitus, serum CA19-9 levels may exhibit a marginal increase (4–7). The current study presents a case of intralobar pulmonary sequestration, which led to upper left abdominal bloating and marked elevation of serum CA19-9 levels. The patient was previously diagnosed with suspected gastrointestinal cancer. This study was approved by the Ethics Committee of Tongji Hospital (Wuhan, China). Witten informed consent was obtained from the patient.

## Case report

A 48-year-old male was admitted to the Tongji Hospital (Wuhan, China) presenting with upper left abdominal bloating and marked elevation of serum CA19-9 levels. The bloating was persistent with no improvement following corresponding treatment for two months. The patient had an acute cough and pain of the left chest one month prior to admission. The laboratory examination results were normal with the exception of the marked elevation of serum CA19-9 levels (790.6 U/ml). The patient was administered with two weeks of antibiotic treatment and the clinical symptoms evidently improved, however, the serum CA19-9 levels remained high (703.3 U/ml). The patient’s doctor suspected hepato-biliary-pancreatic disease and the individual was referred to the Tongji Hospital to determine the cause of the elevated serum CA19-9 levels.

**Figure 1:**
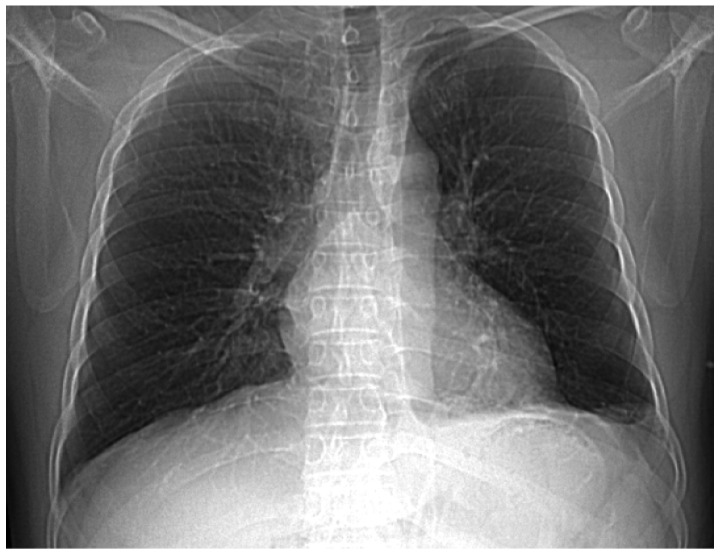
Chest X-ray film on admission showed a dense shadow cord strip in the left lower region.

**Figure 2:**
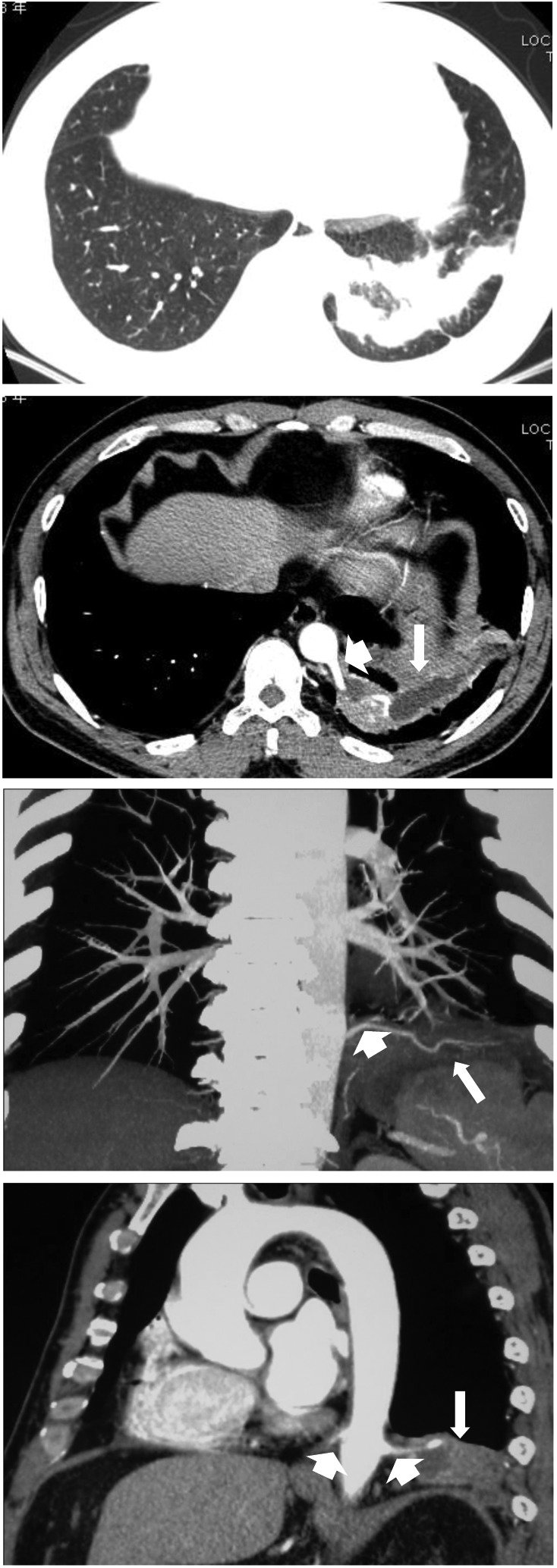
Chest high-resolution computed tomography scanning and 3D image reconstruction showed a mass in the left lower lobe and two anomalous arteries (short arrow) arising from the descending thoracic aorta and extending into the sequestered lung (long arrow).

**Figure 3:**
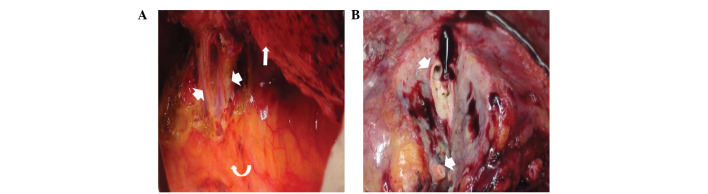
(A) Intraoperative view of two aberrant arteries (short arrows) arising from the thoracic aorta (curved arrow) and entering the left lower lobe basal segment (long arrow). (B) Postoperative view of consolidated lung tissue and aberrant arteries (short arrows).

**Figure 4:**
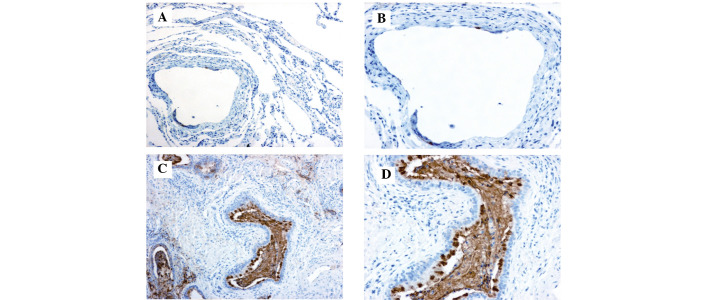
Immunohistochemical staining demonstrated (A and B) weak staining of carbohydrate antigen 19-9 in the normal lung tissue [magnification, (A) ×100, (B) ×200] but (C and D) marked staining in the sequestrated lung tissue, particularly in the mucus of the cysts. [Magnification, (C) ×100, (D) ×200]. The reaction was amplified using the streptavidin-biotin-peroxidase method and diaminobenzidine was used as a chromogen.

**Figure 5:**
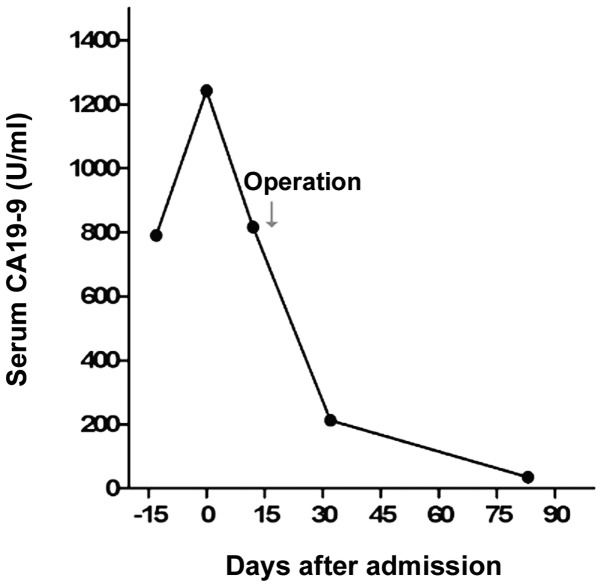
Serum CA19-9 levels prior to and following surgery of intralobar pulmonary sequestration. CA19-9, carbohydrate antigen 19-9.

The patient was a non-smoker and had no family history of pulmonary tuberculosis (TB) or bronchiectasis. However, the patient had pneumonia in 2009, which was cured following antibiotic treatment. The physical examination showed no abnormalities and the laboratory examination revealed a marginal increase in serum γ-guanosine triphosphate (80 IU/l) and serum CA19-9 levels increased to 1,242.85 U/ml. To exclude the possibility of an underlying abnormal malignant lesion, esophagogastroduodenoscopy, endoscopic ultrasonography, colonoscopy, abdominal ultrasonography and computed tomography (CT) were performed with no specific abnormalities identified. A chest X-ray that was performed on admission of the patient to hospital showed a dense shadow cord strip in the left lower region (Fig. 1). In addition, chest high-resolution CT scanning and 3D image reconstruction further indicated anomalous arteries arising from the descending thoracic aorta (Fig. 2). Pulmonary sequestration was diagnosed, and surgery confirmed the presence of two aberrant arteries arising from the thoracic aorta and entering the left lower lobe basal segment. The sequestrated lung was consolidated and tightly connected to the diaphragm (Fig. 3). A left lower lobectomy was performed and the postoperative pathological observations were consistent with intralobar pulmonary sequestration. Immunohistochemistry staining using a monoclonal antibody against human CA19-9 (Maixin Biotechnology, Fuzhou, China) demonstrated marked positive staining for CA19-9 in the ciliated cylindrical epithelia, alveoli and particularly in the mucus of the cysts (Fig. 4). Following pulmonary resection, the symptom of bloating improved and the serum CA19-9 levels rapidly decreased to within the normal range (34.5 U/ml; Fig. 5).

## Discussion

CA 19-9 is valuable as a serum marker for cancers of the biliary, pancreatic and gastrointestinal tracts. However, elevation of serum CA19-9 levels may also occur in the following benign conditions: i) Increased CA19-9 production due to inflammation or proliferation of non-cancerous tissues, such as pancreatitis, pancreatic cysts, cholangitis, bronchial cysts, bronchiectasis and ovarian cysts; ii) CA19-9 discharge pathway obstruction caused by pancreatic or cholangial duct stenosis due to gall stones and papillitis; and iii) malfunction in CA19-9 metabolism, such as chronic hepatitis, chronic glomerulonephritis and diabetes mellitus (8). In the current study, the patient presented with upper left abdominal bloating and marked elevation of serum CA19-9 levels and was diagnosed with intralobar pulmonary sequestration. Immunohistochemistry demonstrated marked positive staining for CA19-9 in the ciliated cylindrical epithelia, alveoli and particularly in the mucus of the cysts. The serum CA19-9 levels rapidly decreased to normal range following surgery. Furthermore, in the present case, no malignant signs were identified in the choledochal, pancreatic or gastrointestinal tracts. These results indicated, in the present case, that CA19-9 was produced in the sequestrated lung and released into the blood via an unknown mechanism.

Pulmonary sequestration is a rare malformation characterized by the presence of lung tissue with abnormal or absent communication with the bronchi. Pulmonary sequestration is classified into the following two types: Intralobar pulmonary sequestration, in which the sequestered section of the lung lies with the normal pulmonary visceral pleura; and extralobar pulmonary sequestration, where the pulmonary tissue is surrounded by the pleura of the lesion itself (9). For the current study, 44 patients that were diagnosed with pulmonary sequestration by surgery at the Tongji Hospital between 2003 and 2012 were reviewed (Table I). There was an approximately equal distribution observed between the genders, and five children and 39 adults were included. The average age of the children that underwent surgery was five years, ranging between two months and 10 years. Among the adult population, the average age was 35 years, ranging between 13 and 59 years. The predominant clinical symptoms of pulmonary sequestration were coughing (50%), fever (25%), hemoptysis (22.7%), expectoration (18.2%), chest tightness (15.9%) and thoracic pain (11.4%). Notably, nine patients were asymptomatic and the majority were identified during a general check up. In total, 38 of the 44 cases of sequestration were intralobar and the other six were extralobar. Of the six patients with extralobar sequestrations, two patients exhibited diaphragmatocele concurrently, however, intralobar sequestrations were not found to be associated with other diaphragmatic or cardiopulmonary anomalies. The sequestrated lung predominantly appeared in the left (65.9%) and right (22.7%) lower lobes; only two cases were identified in the right upper lobe and three occurred in multiple lobes. Sequestrated lung is frequently infected with various bacteria and occasionally it exhibits secondary infection with uncommon pathogens, such as *Mycobacterium* TB and fungus. Examination of the serum CA19-9 levels is seldom advised for suspected pulmonary sequestration patients. Of the 44 patients, the serum CA19-9 levels were detected by chance in three patients prior to surgery. One patient, out of the three, was found to exhibit elevated serum CA19-9 levels (159.2 U/ml), which decreased to within the normal range following surgery.

To the best of our knowledge, the current study is the first report regarding the digestive symptom as well as elevated serum CA19-9 levels caused by pulmonary sequestration. Although comparable cases have been previously reported in Japan and Korea (10–15), the exact mechanism of the condition remains a controversial subject. Previously, Yagyu *et al* (16) inferred that CA19-9 may be synthesized and secreted by normal bronchial epithelial cells, and gradually accumulates in the sequestrated lung with no congestion in the normal bronchial tree. In the current case, immunohistochemistry demonstrated weak staining of CA19-9 in the normal bronchial epithelia, however, marked staining was identified in the sequestrated lung tissue, particularly in the mucus of the cysts. In the present study, a cyst fluid culture was not performed to exclude possible pathogens; however, the results were generally consistent with the viewpoint of Ambiru *et al* (12) that CA19-9, which is concentrated in the sequestrated lung, may transfer into the blood through the injured mucosa of the cyst walls. Furthermore, we predict that the diaphragm, stimulated by the consolidated lung, may lead to the left upper abdominal bloating.

In conclusion, as patients that are diagnosed with pulmonary sequestration may also show normal serum CA19-9 levels (Table I), further basic studies regarding the mechanism of CA19-9 increase in pulmonary sequestration are required. Detecting the level of serum CA19-9 in patients that are diagnosed with pulmonary sequestration may be useful to investigate the correlation between, and mechanism of, serum CA19-9 levels and pulmonary sequestration. In addition, to avoid potential diagnostic pitfalls, it is important for digestive physicians to be aware of the respiratory diseases that are associated with elevated serum CA19-9 levels.

## Figures and Tables

**Table I:** Analysis of 44 cases of pulmonary sequestration surgical procedures at the Tongji Hospital between 2003 and 2012.

Clinical feature	n
Gender	
Male	24
Female	20
Sequestration type	
Intralobar	38
Extralobar	6
Location	
Single lobe	
Left lower lobe	29
Right lower lobe	10
Right upper lobe	2
Multiple lobes	3
Associated diseases	
Intralobar	0
Extralobar (diaphragmatocele)	2
Serum CA19-9 level	
Elevation	1
Normal	2
Not detected	41
Secondary infection	
*Mycobacterium* tuberculosis	1
Fungal	4
Symptom	
Cough	22
Fever	11
Hemoptysis	10
Expectoration	8
Chest tightness	7
Thoracic pain	5
Wheezing	1
Anhelation	1
Abdominal distension	1
Asymptomatic	9

CA, carbohydrate antigen.
